# Replies to Queries on Vaccination and Small-Pox

**Published:** 1828-03

**Authors:** W. Fergusson

**Affiliations:** Surgeon Roy. Af. C. C.


### VACCINATION IN AFRICA.

On the departure of Lieutenant James Holman, R.N. the celebrated blind traveller, for Africa, I begged the favor of him to procure for me some authentic information respecting the present state of small-pox and vaccination in the Negro country, and I supplied him with a few queries calculated to direct his inquiries. He has just transmitted to me the accompanying communication from W. Fergusson, Esq. surgeon of the Royal African Colonial Corps, which appears to me to possess sufficient interest to merit a place in the pages of your excellent Journal.

I am your obedient servant,

GEORGE GREGORY.

*London; December* 19, 1828.

### *Replies to Queries on Vaccination and Small-Pox.*
### By W. Fergusson, Surgeon Roy. Af. C. C.

1st. Is vaccination generally practised among the infant negro population?

2d. Whence do they derive their stock of lymph?

3d. What is the degree of confidence placed in it?

Vaccination is not at all practised among the negro population by native vaccinators; it is, however, practised among certain branches of the negro population by European surgeons. The negro population of Sierra Leone consists of Nova Scotian and Macroon settlers, liberated Africans, and several of the aboriginal African tribes, such as Limmaners, Mandingoes, Soosoos, Sherbros, &c. &c. &c. The three first mentioned of these branches of the negro population, having greater intercourse with Europeans, are better acquainted with European customs, and have, of course, imbibed more of European notions and prejudices on such subjects as the one now under consideration, than the aboriginal inhabitants of this part of Africa: vaccination therefore is, and has been, practised among them to a considerable extent; the stock of lymph being derived from, and kept up by frequent renewal from England. That their confidence in it as a measure preventive of small-pox is great, I judge from the anxiety which they show, and the eagerness which they manifest to have their children vaccinated, when small-pox is raging round them; while, under ordinary circumstances, and when their fears have been lulled by the absence of this fatal epidemic, (an absence which they well know is probably but temporary,) they exhibit such an unaccountable apathy regarding vaccination, that a stranger might well suppose they had no faith in it at all as a prophylactic measure. Notwithstanding this, I believe they have great confidence in it, although, from circumstances to which I shall presently allude, that confidence has, I believe, declined considerably.

4th. How soon does the areola arrive at its greatest height in these countries?

The areola surrounding the vaccine vesicle is, I think, at its greatest height about the eleventh or twelfth day after vaccination, if the lymph used has been genuine.

5th. Does small-pox prevail there?

6th. Does small-pox prevail there after vaccination?

Small-pox prevails occasionally, and there are instances of its having occurred even in a confluent form after vaccination. One genuine instance of this kind came under my notice in the year 1824, in the person of a liberated African girl, of about sixteen years of age. Vaccination had been performed in this case by the late Dr. Nicol, deputy inspector of hospitals, and was considered satisfactory. The case proved confluent. The secondary fever was accompanied by severe diarrhœa, which carried off the patient about the thirteenth day. Another well-authenticated instance of the same fact occurred in the early part of the present year, in the family of a respectable Nova Scotian settler. Other cases of a similar nature have been reported by the inhabitants; but I do not consider that in these cases the proofs of a pure previous vaccine disease have been satisfactorily established. When vaccination has been carried on for some time from the same stock of lymph, the disease is apt to degenerate and become spurious; from which cause we require a frequent renewal of lymph from England, in order to keep it in continuous and successful operation. The spurious disease, on the fifth day, generally shows itself in the form of a small globated papula; on the eighth day, it presents sometimes an ash-coloured pustule, containing purulent matter; at other times, and less frequently, a brown-coloured scab, having a small quantity of purulent matter under it, capable of producing, by inoculation, a disease similar to itself. The great prevalence of a disease among the negro population called *Craw craw* is considered as materially influencing that change in the properties of the pure vaccine lymph which has been just noticed. That apathy and indolence, of which I have already accused the negro population, leads them to consider the appearance of disease in the arm after vaccination as the test of safety from small-pox. Great as the difficulty sometimes is in getting them to bring forward their children for vaccination, it is still greater to procure the necessary examinations in its progress and maturation. The mere appearance of disease in the arm is supposed to carry along with it immunity from small-pox; and, on the occurrence of the epidemic at an after period, it may be easily foreseen how wretchedly and how fatally this confidence in the spurious disease may be misplaced. I, therefore, do not consider that, in all the cases spoken of among the inhabitants as cases of small-pox occurring after vaccination, there existed satisfactory proofs of the patient's having previously undergone the genuine vaccine disease; yet I am sorry to say that, from such occurrences as these, vaccination has rather lost ground in the opinion of the negro population.

7th. Is small-pox an increasing malady?

Small-pox is not an increasing malady: it is generally introduced here from the slave cargoes of vessels detained by the squadron, and sent here for adjudication. Were this source of its renewal removed, I am persuaded that small-pox would, in the course of a few years, be almost unknown in this part of Africa.

8th. Can the vaccine virus be retained on points and glasses, so as to be fit for use?

The vaccine lymph, if taken on points, will not retain its power seven days in this country. This observation is established by repeated trials. If taken on glasses, I would not be disposed to depend on its activity when kept longer than fourteen or sixteen days, though I have known it sometimes to retain its original properties for four or five weeks. If preserved in glass bulbs hermetically sealed in the manner practised by the National Vaccine Institution, I have known its properties unimpaired after keeping for three months. Repeated trials have convinced me of the excellence of this mode of preserving the vaccine lymph, and I believe it to be the best and surest that has been yet devised of transmitting the lymph from England to tropical countries: next to this method, I believe the crusts have proved most successful.

9th. Are there periodical vaccinations of large districts, or is each child vaccinated soon after its birth: if the latter, how soon?

The practice in these cases is, as long as the vaccine lymph continues to produce a genuine disease, to keep it up by the weekly vaccination of all comers. Children are rarely vaccinated under four weeks old, but there is no rule observed on this head.

10th. What sort of scars are usually left in the arms?

The scar bears the shape of the original vesicle, and is slightly depressed below the surface of the surrounding skin. The surface of the scar is marked by a number of small depressions of various shapes, corresponding, I believe, with the cells in the original vesicle.

11th. Is vaccination in hot countries attended with feverish symptoms? If it is, on what day do they begin?

Vaccination is sometimes in this country attended with feverish symptoms; but, in the most marked cases, so far as I have seen, these symptoms have been so slight as almost to escape common observation. I have not remarked on what day they begin.

12th. Is vaccination ever followed by any eruptions?

I have seen only one case of this: an eruption appeared on the sixth day after unsuccessful vaccination: it was diffused over the whole body, and is now in progress.

*Sierra Leone; September* 24, 1827.

N.B.—The case alluded to in the last of the above replies was in the first instance a papular eruption, the base of each papula being surrounded by an inflamed ring. The eruption was thickest on the thorax and on the arms. In its progress the eruption became pustular, the pustules being in circumference about half the usual size of the vaccine vesicle. On the twelfth day, the crusts had dropped from some of the smaller pustules; and, by the seventeenth day, they had all dropped off, leaving a mark, but not in any manner pitted, and which I do not think promises to be permanent.

---

